# Ciliary Genes in Renal Cystic Diseases

**DOI:** 10.3390/cells9040907

**Published:** 2020-04-08

**Authors:** Anna Adamiok-Ostrowska, Agnieszka Piekiełko-Witkowska

**Affiliations:** Department of Biochemistry and Molecular Biology, Centre of Postgraduate Medical Education, 01-813 Warsaw, Poland

**Keywords:** primary cilia, ciliopathies, renal cell cancer, RCC, polycystic kidney disease, nephronophthisis, *PKD1*, *PKD2*, *VHL*, *OFD1*

## Abstract

Cilia are microtubule-based organelles, protruding from the apical cell surface and anchoring to the cytoskeleton. Primary (nonmotile) cilia of the kidney act as mechanosensors of nephron cells, responding to fluid movements by triggering signal transduction. The impaired functioning of primary cilia leads to formation of cysts which in turn contribute to development of diverse renal diseases, including kidney ciliopathies and renal cancer. Here, we review current knowledge on the role of ciliary genes in kidney ciliopathies and renal cell carcinoma (RCC). Special focus is given on the impact of mutations and altered expression of ciliary genes (e.g., encoding polycystins, nephrocystins, Bardet-Biedl syndrome (BBS) proteins, ALS1, Oral-facial-digital syndrome 1 (OFD1) and others) in polycystic kidney disease and nephronophthisis, as well as rare genetic disorders, including syndromes of Joubert, Meckel-Gruber, Bardet-Biedl, Senior-Loken, Alström, Orofaciodigital syndrome type I and cranioectodermal dysplasia. We also show that RCC and classic kidney ciliopathies share commonly disturbed genes affecting cilia function, including *VHL* (von Hippel-Lindau tumor suppressor), *PKD1* (polycystin 1, transient receptor potential channel interacting) and *PKD2* (polycystin 2, transient receptor potential cation channel). Finally, we discuss the significance of ciliary genes as diagnostic and prognostic markers, as well as therapeutic targets in ciliopathies and cancer.

## 1. Structure and Function of Cilia

**Figure 1:**
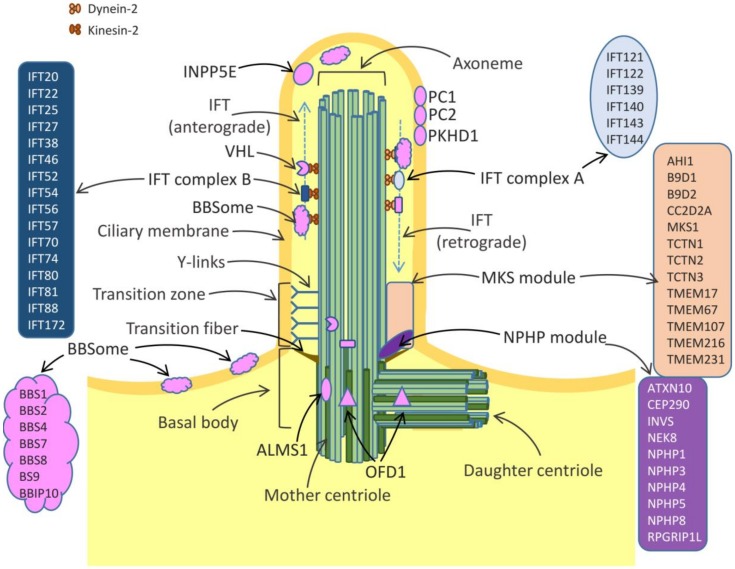
The structure of primary cilium. The axoneme is composed of nine pairs of microtubules, anchored in the cell by the basal body. The latter is a modified centriole, consisting of nine triplets of microtubules. The mother centriole plays a key role in ciliogenesis, recruiting the molecules required for axoneme elongation. The daughter centriole results from duplication of mother centriole during S phase [4]). The arrows indicate the key structural cilium elements (the axoneme, transition zone and basal body) as well as proteins involved in kidney ciliopathies and renal cell carcinoma (RCC) [4,5,17,18].

Discovered almost coincidentally in 1676 by Anton van Leeuwenhoek, cilia have been ignored by the scientific community until the 20th century when electron microscopy and immunocytochemistry techniques allowed the fine description of the ciliary structure and composition [1,2]. Nowadays, cilia are defined as microtubule-based organelles which protrude from the cell apical surface and anchor to the cytoskeleton. The three key important structures of cilia include the axoneme, the basal body and the transition zone (Figure 1). The axoneme is a ring of microtubules, which connect the cilium tip with its base and provide proper trafficking of proteins mediated by intraflagellar transport (IFT) machinery. The axonemes of all cilia are built of nine pairs of microtubules. Motile cilia possess an additional pair of central microtubules, which are absent from the immotile primary cilia [3]. The basal body is attached to the plasma membrane by transition fibers and provides a platform anchoring the cilium in the cell body. Transition zone (TZ) is formed by protein complexes termed the nephronophthisis (NPHP) module and the Meckel-Gruber syndrome (MKS) module. The TZ structure is stabilized by specific structures called y-links, which together with transition fibers control the entrance and exit of proteins to/from the cilia [4,5]. Cilia are devoid of protein synthesis systems, therefore all ciliary proteins are synthesized in the cell body and transported via IFT. The IFT system is composed of multiple proteins, of which kinesin-2 and dynein 2 play key roles in trafficking of the cargo proteins from the cell body to the cilium top (the anterograde transport) and from tip of cilium towards the cell body (the retrograde transport) (Figure 1).

Cilia play many important roles during development of cells, tissues and organs. Primary cilia act as sensory organs involved in transmission of signals from the extracellular environment into cells, by triggering crucial signaling pathways including Wnt, Planar Cell Polarity (PCP) and Hedgehog pathways [6]. They detect different stimuli such as fluid shear, mechanic deformation (movement, vibration, touch), light or odorants. In response to fluid movements, primary cilia of renal epithelium initiate an intracellular calcium signaling [7]. Olfactory sensory cilia are responsible for odorant detection through their G-protein coupled receptors. The invertebrate chordotonal organ is equipped with cilia of sensory neurons which can change their shape, thereby initiating rapid electrical response via ion channels located at the axoneme [8,9].

Structural or functional impairments of primary cilia caused by mutations in ciliary genes lead to dysregulation of signals transduction or inability to response to stimuli. For instance, Wnt signaling pathway required for the growth of renal tubules in mouse is disrupted by mutations of *Invs*, a ciliary gene encoding inversin [10]. In humans, *INVS* mutations cause nephronophthisis [11]. Mutations in *PKD1* and *PKD2* genes, encoding polycystins expressed at kidney primary cilia disrupt formation of cation channel, impairing Ca^2+^ influx and detection of fluid flow. Finally, mutations that disrupt cilia formation disable signaling mediated by Pdgfrα (platelet-derived growth factor receptor alpha) [12].

Motile cilia generate efficient fluid movement required for proper development and function of different tissues and organs. For instance, cilia of the female reproductive tract are involved in the egg transportation in the oviduct, while in the brain ventricles they ensure proper circulation of cerebrospinal fluid. In vertebrate, respiratory tract cilia are responsible for lung clearance from the inhaled particles [13]. Interestingly, these cilia play an additional mechanosensation role by expressing sensory bitter taste receptors [14]. Finally, nodal cilia by their clockwise beating activity establish left-right body asymmetry [15]. The most common defects in motile cilia structure are lack of dynein arms, radial spokes or central pair of microtubules, which impair cilia beating activity or frequency. Dynein arms defects are caused, among others, by mutations in genes encoding heavy chain subunits of dynein, *DNAH5* and *DNAH11* [16]. One of the clinical symptoms of mutated dynein is loss of ciliary function in respiratory tract resulting in chronic respiratory infections. Impaired motile cilia in the female reproductive tract affect fertility, while in brain ventricles their aberrant functioning leads to hydrocephalus [13]. In the mouse node, defective cilia abolish leftward fluid flow and cause asymmetric gene expression and disturbed morphogenesis. Finally, any abnormalities in cilia morphology such as shortened or elongated length, abnormal shape, but also increase or decrease of their number have functional consequences which impair cellular homeostasis.

The biomedical relevance of primary cilia was disclosed through the discovery of cyst formation induced by perturbed function of ciliary protein ift88 in the mouse kidney [19,20]. However, single cilium is composed of hundreds proteins responsible for their proper structure and function. These ciliary proteins contribute to the cellular response to signals from different pathways, including the Hedgehog, Planar Cell Polarity (PCP), Platelet-derived growth factor (PDGF), fibroblast growth factor (FGF) and VHL/GSK3β (the von Hippel–Lindau tumor suppressor/Glycogen synthase kinase 3 beta) pathways [6,21]. The clinical features of ciliopathies are commonly shared by pleiotropic developmental disorders [22], including abnormalities in neural tube closure, polydactyly, liver diseases, retinal degeneration, anosmia, cognitive defects, obesity, randomization of the left-right body axis and cystic kidneys [23]. Here, we discuss the kidney ciliopathies by characterizing the ciliary genes involved in their pathology.

## 2. Role of Cilia in Renal Diseases

Under normal conditions cilia of nephron cells detect the flow of fluid through the tubule lumen [24]. Defective fluid flow sensing triggers formation of cysts [25], leading to ciliopathies, including polycystic kidney disease (PKD), Nephronophthisis (NPHP), Joubert syndrome, Meckel-Gruber syndrome, Bardet-Biedl syndrome, Senior-Loken syndrome (SLS), Alström syndrome (AS), Orofaciodigital syndrome type I (OFD) and Cranioectodermal dysplasia (CED). Remarkably, these different ciliopathies share common mutated genes encoding key cilia proteins. Below we discuss genes most frequently responsible for characteristic symptoms of ciliopathies.

### 2.1. Polycystic Kidney Disease

PKD ciliopathies include the autosomal dominant (ADPKD) and autosomal recessive (ARPKD) polycystic kidney disease. Although these PKD subtypes differ by their inheritance patterns and the age-dependent occurrence (with ADPKD presenting mainly in adults and ARPKD occurring mainly in early childhood), both share similar clinical and molecular features [26]. The characteristic clinical feature of PKD is hypertension resulting from the activation of intrarenal renin-angiotensin-aldosterone system. The cystic epithelial and tubular cells produce renin, angiotensinogen and angiotensin II which are secreted into cystic fluid [27,28]. ADPKD, affecting one in 400-1000 individuals, is caused by mutations in two genes, *PKD1* or *PKD2* [29]. Most ADPKD cases (85%) are associated with mutations of *PKD1* gene (located at 16p13.3 chromosome). The mutations in *PKD2* (located at 4q22.1 chromosome), found in the residual 15% of PKD patients, lead to milder kidney polycystic disease symptoms compared with patients with *PKD1* mutations [30,31].

**Figure 2:**
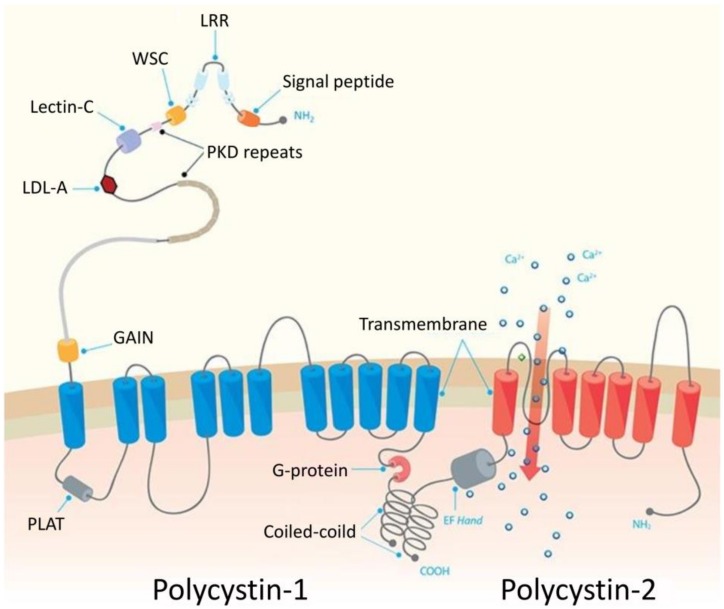
The structure of polycystins. LRR: leucine-rich repeats; WSC: cell wall integrity and stress response component domain; Lectin C: lectin C type-3 domain; LDL-A: low-density lipoprotein-A domain, PKD: polycystic kidney disease repeats; GAIN: G-protein-coupled receptor (GPCR) autoproteolysis-inducing domain, PLAT: Polycystin-1, Lipoxygenase, Alpha-Toxin domain. The Figure reprinted and modified with permission from [45] under Creative Commons Attribution 4.0 International (CC BY 4.0).

*PKD1* and *PKD2* encode polycystin 1 (PC1) and polycystin 2 (PC2), respectively. PC1 is a large (~460 kDa) glycoprotein with >3000-amino acid extracellular region, eleven transmembrane domains and a short 197-amino acid intracellular domain (Figure 2). The extracellular N-terminal region mediates PC1 interactions with other extracellular proteins and carbohydrates. Its key elements include leucine rich repeats (LRR), sixteen immunoglobulin-like PKD repeats and receptor for egg jelly (REJ) module, as well as the GPCR autoproteolysis-inducing (GAIN) domain that includes a 50-aa GPCR proteolysis site (GPS) motif [32,33]. The GPS cleavage is indispensable for intracellular PC1 trafficking to the primary cilia and formation of the PC1/PC2 complex, and plays a crucial role in the postnatal maturation of distal nephron morphology. The common feature of both polycystins is cytoplasmic coiled coil domain at a C-terminal through which they interact with each other. Compared to polycystin 1, PC2 is a smaller (~110 kDa) protein with six transmembrane regions (Figure 2) [34]. Both polycystins are highly expressed in the epithelial cells of fetal and adult kidney [35] and their expression can be regulated by the key cellular signaling pathways and transcription regulators, including beta-catenin/TCF and JAK/STAT (Janus kinase/Signal transducer and activator of transcription) pathways targeting *PKD1* [36,37], Sp1 regulating *PKD1* and *PKD2* [38,39] and p53 targeting *PKD1* [40]. Furthermore, the expression of polycystins is regulated at the translational and posttranslational manner. Specifically, far upstream element-binding protein 1 (FUBP1) regulates protein synthesis of PC2 and enhances its stability in a mechanism involving filamin-A [41]. The main role of PC1 and PC2 is formation of a protein complex which acts as a receptor-ion channel in primary cilia (Figure 2). The Ca^2+^ influx is induced by binding of Wnt ligands to the extracellular PC1 domain in the presence of PC2. Although it is known that *PKD1* or *PKD2* mutations disrupt complex formation, reducing channel permeability [42], the exact molecular mechanisms that contribute to the development of ADPKD are poorly understood [29]. Furthermore, recent studies suggested that the pathogenic mechanism of PKD could be independent of ion-channel activity of PC1, which may be irrelevant to the ion currents in the cilium, while PC2 may act as a monovalent cation-selective channel [43]. About 30% of PKD-related PC1 mutations affect the GAIN domain and the neighboring REJ region, suggestive of impaired GPS cleavage in cells of PKD patients. This observation raised the concept of therapeutic strategies aiming in improvement of PC1 proteolytical processing, e.g., by specifically designed chaperoning molecules [32]. The intracellular C-terminal PC1 domain mediates interactions with PC2, enabling the formation of the receptor-channel complex. It is also a binding site for of tuberin, a protein regulating mTOR (mammalian target of rapamycin) kinase activity in a manner dependent on small GTPAse Rheb. It was shown that the mTOR pathway is inappropriately activated in epithelial kidney cells of ADPKD patients and mouse models, suggesting a possible mechanism of pathogenic *PKD1* mutations. Furthermore, treatment of ADPKD transplant-recipient patients with mTOR inhibitor was associated with significant reduction of the size of the endogenous polycystic kidney, indicating the key role of mTOR in ADPKD pathology [44]. PC1 is also proteolytically cleaved at the C-terminal part, leading to the release of three protein fragments of different size which migrate to the nucleus where they interact with transcription regulators [34].

The ARPKD is much less common than ADPKD and occurs in 1:20,000 live births in Caucasian populations. ARPKD is caused by mutations in *PKHD1* gene. The transcription of *PKHD1* is controlled by TCF-2 transcription factor, of which mutations impair *PKHD1* transcription and result in renal cysts [46,47]. *PKHD1*, encoding fibrocystin, known also as polyductin, is a large protein (>4000 amino acids) spanning the ciliary membrane. Fibrocystin is composed of the highly glycosylated N-terminal tail that contains several IPT and IPT-like domains and multiple parallel beta-helix repeats (PbH1), a single transmembrane region, and the C-terminal part with kinase A phosphorylation sites. At a cytoplasmic tail fibrocystin possess ciliary targeting sequence (CTS) which controls trafficking of regulatory proteins to the cilium [48]. The exact function of fibrocystin is unknown. Basing on structural similarities to other proteins, fibrocystin is supposed to act as a cell surface receptor, involved in the regulation of cell-cell adhesion and proliferation. Fibrocystin colocalizes with polycystins and is thought to be involved in microtubule organization and mechano- and chemosensing mechanisms mediated by the cilia [48]. Similarly to PC1 and PC2, the exact mechanisms by which *PKHD1* mutations contribute to the development of PKD are unknown. The second gene involved in ARPKD is *DZIP1L*, encoding DAZ (Deleted in Azoospermia) - interacting protein 1-like, a 767 amino acid protein which localizes to centrioles and the distal end of the basal body, contributing to the maintenance of the TZ diffusion barrier. *DZIP1L* mutations were identified in seven ARPKD patients from four families who lacked mutations in *PKHD1* gene. The pathological significance of *DZIP1L* aberrations was confirmed using mice with chemically induced *Dzip1l* mutations which caused cystic kidney disease [49].

### 2.2. Nephronophthisis

Nephronophthisis (NPHP) is an autosomal recessive, genetically and clinically heterogeneous disorder, which occurs equally in both sexes with incidence ranging from 1:50,000 (Finland, Canada) to 1:1,000,000 (USA) [50,51,52]. NPHP is characterized by fibrosis and cyst formation in the kidney, progressive reduction of kidney size, polyuria and polydipsia [53]. Depending on the time of end-stage renal disease (ESRD) occurrence, NPHP is classified into three different types: the juvenile or type 1 (with ESRD developed at the mean age of 13 years), infantile or type 2 NPHP (ESRD occurring prior to 4 years of age) and adolescent or type 3 NPHP (ESRD at the mean age of 19 years). NPHP patients are offered only supportive therapy, aiming in attenuation of disease progression. In case of terminal kidney failure, the only treatment options remaining are dialysis and kidney transplantation [54,55]. NPHP is associated with mutations in so far 20 identified genes, of which most encode nephrocystins, proteins localizing to the cilium transition zone [54]. Nephrocystins are composed of several domains responsible for protein-protein and protein-DNA interactions, however with no commonly shared protein domains pattern [50]. The ciliary functions of proteins encoded by genes most frequently mutated in nephronophthisis are discussed below.

*NPHP1* encodes nephrocystin 1, a 733 amino acid protein contributing to the formation of NPHP module, localizing to the base of the transition zone and regulating IFT [56]. Nephrocystin 1 is crucial for proper cilia morphology, as indicated by *NPHP1* knockdown in the Madin-Darby canine kidney (MDCK) cells, which results in abnormal cilia formation [57]. Another component of NPHP module is inversin, encoded by *NPHP2/INVS*. In renal cilia, inversin suppresses the canonical Wnt pathway [58] and acts as a switch between the canonical and non-canonical Wnt pathways. Through its interaction with the Planar Cell Polarity (PCP) signaling protein dishevelled (Dsh or Dvl1), inversin establishes cell orientation and maintains tubular architecture in developing nephrons [11]. Mouse *Invs* knockouts develop large cystic kidneys at an early stage [59]. Both the above mentioned proteins (nephrocystin 1 and inversin) interact with nephrocystin 3, encoded by *NPHP3*, which inhibits the canonical Wnt pathway [50,60,61]. *NPHP4* encodes nephrocystin 4 (known also as nephroretinin) localizing to the primary cilium and its basal body. Nephrocystin 4 forms complexes with α-tubulin, nephrocystin 1 and RPGRIP1 [62,63]. *NPHP4* interplays with *NPHP1* and this interaction is crucial for cell-cell and cell-matrix adhesion signaling events [62]. By interactions with PLAS1, PAR6 and PATJ, nephrocystins 1 and 4 regulate organization of renal epithelial cell [57]. *nphp-1* and *nphp-4*, along with other proteins of MKS/BD9/NPHP families, are also involved in the early stages of ciliogenesis in *C. elegans* [64,65]. *NPHP5* encodes nephrocystin 5, a protein composed of two calmodulin binding domains. Calmodulin, nephrocystin 5 and retinitis pigmentosa GTPase regulator (RPGR) form a complex present in cilia of photoreceptors and renal epithelial cells [66]. *NPHP6/CEP290* encodes nephrocystin 6, localizing in the centrosome and cilia. *CEP290* plays a crucial role in ciliogenesis in a mechanism involving GTPase RAB8A in human retinal pigment epithelial cells [67]. CEP290 interacts with PCM1 and CEP72. Under normal conditions, CEP290 binds translation product of McKusick-Kaufman syndrome gene called MKKS. In Bardet-Biedl syndrome (discussed below) *MKKS* mutations result in weakening or even loss of CEP290 interactions [68]. The depletion of *CEP290* reduces ciliary recruitment of BBS proteins required for primary cilia formation in mouse and human cells [69]. In zebrafish model depletion of *nphp5* or *nphp6* leads to pronephric cysts [70]. *NPHP7/GLIS2* encodes transcription factor GLIS2, which localizes to the primary cilia and nucleus. Mouse mutant of *Glis2* shows tubular atrophy and kidney fibrosis [71]. *NPHP8/RPGRIP1L* encodes a centrosomal protein called retinitis pigmentosa GTPase regulator interacting protein 1-like, which interacts with NPHP4, NPHP6 and gamma-tubulin. Several reports demonstrated expression of RPGRIP1L in basal bodies and ciliary axonemes at the base of primary cilia [72,73]. Mouse *Rpgrip1l* knockouts develop abnormal cilia devoid of axoneme in the forebrain neuroepithelial cells [74]. *NPHP9/NEK8* encodes a never in mitosis A-related kinase 8 which forms a complex with inversin, NPHP3 and ANKS6 [75]. Interestingly, NEK8 interacts with ARPKD protein polycystin-2 to regulate ciliary localization of both PC1 and PC2 [76]. Morpholino-induced knockdown of *nek88* in zebrafish results in pronephric cysts formation, while overexpression of human *NEK8* leads to pronephros abnormalities [77].

Nephronophthisis can be also caused by mutations in genes encoding proteins other than nephrocystins, such as *TTC21B*, *CEP164*, *ANKS6*, *CEP83* and *DCD2*. *TTC21B* encodes tetratricopeptide repeat-containing hedgehog modulator-1 (THM1), which is an axonemal protein required for intraflagellar transport. Murine *Ttc21b* through *alien* locus regulates sequestration of IFT proteins such as IFT88 [78]. *CEP164* encodes a centrosomal protein CEP164 required for assembly of primary cilia [79]. Knockdown of *Cep164* in the mouse kidney IMCD3 (mouse kidney inner medullary collecting duct) cells in 3D spheroid growth assays reduces ciliation frequency, while in human RPE1 cells attenuates ciliogenesis [80,81]. In zebrafish, knockdown of *cep164* leads to formation of pronephric tubule cysts [80]. ANKS6 protein, encoded by *ANKS6* gene, contributes to assembly of ciliary proteins required for renal development. Depletion of *anks6* and its interacting proteins, NEK8 and NPHP3, results in formation of pronephric cysts during zebrafish development [75]. *CEP83* (alias *CCDC41*) encodes an 83kDa centrosomal protein which initiates primary cilia assembly by docking ciliary vesicles to mother centriole [82]. *DCDC2* gene encodes a protein including two doublecortin peptide domains. DCDC2 binds tubulin, enhances microtubules polymerization and inhibits canonical Wnt signaling [83,84]. Knockdown of *DCDC2* in kidney cell lines and zebrafish embryos reduced the number of cilia. Interestingly, these defects are recovered after treatment with Wnt inhibitor [83].

### 2.3. Joubert Syndrome

Joubert syndrome (JBTS) is characterized by hypoplasia of the cerebellar vermis with the characteristic ‘molar tooth sign’, developmental delay, neonatal breathing abnormalities, muscular hypotonia, ataxia and cyst formation in the kidneys. The syndrome affects 1 in 80,000–100,000 newborns and is associated with mutations in 35 ciliary genes [85]. Defects in genes associated with Joubert syndrome are often the main causes of other ciliopathies such as Nephronophthisis or Meckel-Gruber syndrome. Joubert syndrome was classified into 35 subtypes of which most are caused by mutations linked with kidney impairments. For instance *AHI1*, a gene associated with Joubert syndrome type 3 (JBTS3; MIM 608629), encodes jouberin, a protein composed of WD repeats and an SH3 domain. *AHI1* plays an important role in targeting ciliary membrane proteins to the growing cilium [86]. Jouberin interacts with RAB8 GTPase, thereby regulating vesicle transport and cilium formation. *Ahi1* knockdown in mouse leads to impaired ciliogenesis and diminished kidney with characteristics of nephronophthisis [86]. The maintenance of the proper primary cilium structure is also regulated by *INPP5E* encoding inositol 1,4,5-trisphosphate (InsP3) 5-phosphatase. INPP5E (JBTS1; MIM 613037) controls growth and proper formation of cilia in a mechanism involving phosphoinositide 3-kinase (PI3K) and platelet-derived growth factor receptor A (PDGFRA) [87]. Proper INPP5E targeting to cilia is dependent on interactions with prenyl-binding protein phosphodiesterase 6D (PDE6D) and ARL13B (JBTS8; MIM 608922), a small GTPAse called ADP-ribosylation factor-like 13 (ARL), which promotes the release of INPP5E from PDE6D [81]. *ARL13* is expressed in cilia of distal renal collecting duct and its missense mutations found in JBTS patients disrupt interaction of ARL13B protein with INPP5E, disabling cilia targeting of the latter [81,88]. Inactivation of *Arl13b* in zebrafish results in formation of renal cysts and curved tail, the specific features reflecting impaired cilia [89]. On the other hand, cilial localization of ARL13B is regulated by CSPP1 (JBTS21; MIM 615636), a centrosome spindle pole-associated protein-1 involved in cell cycle progression and spindle organization. *Cspp1* knockdown in zebrafish results in reduced *arl13b* ciliary localization and formation of pronephric cysts [90]. JBTS is also associated with mutations in *ARL3* (JBTS35; MIM 61816), encoding ADP-ribosylation factor-like 3, crucial for axoneme formation by cargo displacement of lipidated proteins in the cilium [91]. Mice devoid of *Arl3* develop abnormal renal epithelial tubule structures and cysts [92]. Another gene involved in JBTS pathology is *CEP41* (JBTS15; MIM 614464), which encodes centrosomal protein regulating tubulin glutamylation, thereby contributing to axonemal formation [93].

### 2.4. Meckel-Gruber Syndrome

Meckel-Gruber syndrome is linked with a wide spectrum of pathological features, including renal cystic dysplasia. The global incidence rate of Meckel-Gruber syndrome is 1 per 135,000 live births [94]. This inherited recessively ciliopathy is associated with mutations in multiple genes, encoding the protein components of the MKS module (including B9D1, B9D2, CC2D2A, MKS1, TCTN2, TMEM216, TMEM67, TMEM107 and TMEM231) as well as nephrocystins (e.g., NPHP3, NPHP6, NPHP8). MKS1, B9D1 and B9D2 belong to the small family of B9 domain-containing proteins and provide docking sites at the ciliary gate that prevents unwanted diffusion of membrane proteins into primary cilia [65]. Mutations in *Mks1* gene associated with Meckel-Gruber syndrome perturb Hedgehog signaling in a mouse model [95]. MKS1 binds meckelin, encoded by *MKS3/TMEM67.* Knockdown of *Mks1* and *Mks3* in mouse inner medullary IMCD-3 cells inhibits migration of centrioles to the apical membrane [96]. Tammachote and co-workers suggested that MKS1 and meckelin regulate length and the appropriate number of cilia by modulating centrosome duplication. Indeed, *Mks3*-null mouse embryos develop cysts and reduced number of renal cilia [97]. Meckelin contributes to cilia formation by interacting with filamin A. Loss of meckelin or filamin A leads to abnormal basal bodies positioning, aberrant remodeling of actin cytoskeleton, deregulation of RhoA activity and hyperactivation of Wnt signaling [98]. Furthermore, meckelin interacts with TCTN1 and TCTN2 belonging to the tectonic proteins family [99]. Together with TMEM216, TMEM67, CEP290, B9D1 and CC2D2a, these proteins are localized to the transition zone, where they control ciliogenesis and ciliary membrane composition. In mouse embryos *Tctn1* or *Tctn2* knockouts result in defective cilia, which fail to elongate the axoneme [99].

Another group of molecules that localize to the transition zone are proteins of the TMEM (transmembrane) family. In particular, TMEM216 is required for the assembly and proper function of cilia. *TMEM216* knockdown leads to hyperactivation and mislocalization of Rho GTPase, a protein crucial for basal bodies docking at the apical surface of plasma membrane [100]. In mouse IMCD3 cells, *Tmem216* knockdown results in reduction of cilia length and other ciliogenesis defects [101]. The other TMEM proteins involved in cilia development and functioning include TMEM107, predicted to be essential for cilia formation and signaling in embryonic tissues [102] and *Tmem231* of which knockdown in mouse cells interferes with Sonic Hedgehog signaling by preventing the movement of Smo into the ciliary membrane. Inactivation of *TMEM231* also leads to delayed ciliogenesis and cilia growth due to the absence of diffusion barrier [103]. The murine transition zone proteins TCTN1 and TCTN2 interact also with CC2D2A [99]. In *C. elegans* the latter forms a transition zone complex together with *nphp-1*, *nphp-4*, *tmem-67*, *mks-1*, *b9d1*, *b9d2* and *rpgrip-11* [65].

### 2.5. Bardet-Biedl Syndrome

Bardet-Biedl syndrome (BBS), reported by Laurence and Moon in 1866, is associated with gonadal abnormalities, retinal degeneration, mental retardation, obesity, diabetes, polydactyly, situs inversus and chronic renal failure in children. In some cases syndactyly, brachydactyly and/or clinodactyly may be present. Bardet-Biedl syndrome has a prevalence of 1 in 125,000 [104] and is caused by mutations in *BBS* genes, but also in genes associated with other ciliopathies, such as *NPHP6*, *NPHP11*, *MKS1*, *SDCCAG8*, *LZTFL1*, *BBIP1* and *IFT27.* BBS proteins localize to the basal body and the axoneme of cilia, and contribute to the formation of the BBSome, a multiprotein complex responsible for transportation of intracellular vesicles to the base of cilia. Within the complex, BBS1 mediates interaction with the guanine nucleotide exchange factor for small G protein RAB8; BBS5 participates in phospholipids binding, while BBS9 is a BBSome organizing subunit [105]. The other BBSome components include BBS2, BBS4, BBS7, BBS8 and BBS9. The interaction between *Bbs* genes and *Vangl2*, a component of Planar Cell Polarity (PCP) signaling pathway was demonstrated in mouse and zebrafish models [106].

The BBS proteins that do not form BBSome complex include BBS6, BBS10 and BBS12. BBS6 (or *MKKS)* is required for retrograde cellular transport and proper cilia functioning. The zebrafish *bbs6 (mkks*) morphants have shorter cilia of their Kupffer vesicles [107]. BBS10 and BBS12, localize to the primary cilia of human preadipocytes, where they carry Wnt and Hedgehog receptors [108].

*ARL6* gene encodes a protein that binds to the BBSome and BBIP1. Due to ARL6 recruiting activity BBSome polymerizes into an electro-dense planar coat which allows protein transport to the ciliary membrane. Interestingly, the BBSome recruitment by ARL6 depends on GTP binding, but not on ARL6 activity. Depletion of *ARL6* in human RPE cells leads to BBSome mislocalization [109].

*CCDC28B* encodes a coiled-coil domain-possessing 28B protein of which knockdown in human telomerase reverse transcriptase (TERT)—immortalized retinal pigment epithelial cells leads to the reduced number and length of cilia. Furthermore, *ccdc28b* zebrafish morphants show perturbed pronephron ciliogenesis [110].

*LZTFL1* encodes a leucine zipper transcription factor-like 1, transiently associated with BBSome through interaction with BBS9. LZTFL1 protein plays a role in BBSome trafficking, but not assembly. Depletion of *Lztfl1* in mouse alters Hedgehog signaling [111].

*BBIP1* encodes BBS protein complex-interacting protein 1 involved in ciliary membrane growth and stabilization of cytoplasmic microtubules by controlling their acetylation and polymerization [112]. Depletion of *BBIP1* in RPE cells increases the frequency of centrosome splitting in interphase cells, while *bbip1* knockdown in zebrafish results in cystic dilations of the pronephrons. The cilia of pronephrons are shorter and fail to maintain parallel orientation [113].

*IFT27* and *IFT74* genes encode proteins involved in intraflagellar transport. IFT27 is a small G protein, which participates additionally in cell division, while IFT74 binds and transports tubulin within the cilia [114,115]. *Ift74* knockdown in zebrafish leads to renal abnormalities consistent with ciliopathy [116].

### 2.6. Senior-Loken Syndrome

Senior-Loken syndrome (SLS) is an autosomal recessive disease with the main features of nephronophthisis, but associated with retinitis pigmentosa. The prevalence of nephronophthisis is estimated to be 1 in 100,000, with 1 in 10 affected individuals having retinal dysfunction, constituting Senior-Loken syndrome. SLS is caused by mutations in *NPHP1*, *NPHP4*, *NPHP5*, *NPHP6*, *SDCCAG8*, *WDR19/IFT144* and *TRAF3IP1* genes [66].

*SDCCAG8* encodes serologically defined colon cancer antigen, which localizes to centrosomes and cell-cell junctions in mammalian renal epithelial cell line, and interacts with OFD1, NPHP5 and ninein. Zebrafish morphants for *sdccag8* develop multiple cysts in their kidneys [117].

*TRAF3IP1* encodes a subunit of IFT-B complex which mediates anterograde transport in cilia (ITF54 protein). Knockdown of *TRAF3IP1* homolog *elipsa* in zebrafish leads to pronephric cysts, while mouse mutants show ciliary assembly defects and diminished expression of Shh reporter [118,119].

### 2.7. Alström Syndrome

Described for the first time in 1959, Alström syndrome (AS) occurs rarely, with incidence 1-9 cases per 1,000,000 individuals. Renal failure is often observed in AS patients, however the main symptoms of this disease are the progressive cone-rod dystrophy leading to blindness, sensorineural hearing loss, childhood obesity associated with hyperinsulinemia and type 2 diabetes mellitus. Alström syndrome patients rarely live beyond 50 years of age and often require dialysis or kidney transplantation [120,121,122]

Alström syndrome is caused by mutations in *ALMS1* gene. ALMS1 protein is a component of centrosome and participates in pericentrioral material (PCM) assembly [121]. Mouse model of Alström syndrome with truncated ALMS1 protein shows cilia loss from kidney proximal tubules [123]. *Alms1* -/- mice accumulate intracellular vesicles in the inner segments of photoreceptors [124].

### 2.8. Orofaciodigital Syndrome

Mutations of *OFD1* gene cause Orofaciodigital syndrome type 1 (OFDI), distinguished from the other Orofaciodigital syndromes by X-linked dominant inheritance and cystic kidney disease. The common phenotypic features of all OFD syndromes include malformations of the face, oral cavity and digits. Initially, OFD syndromes were classified into 13 subtypes, characterized by different clinical phenotypes. In 2017, novel classification, based on combined clinical and molecular data, reduced the number of OFD syndromes to the three main subtypes: OFDI (associated with mutations in *OFD1*), OFDIV (linked with mutations in *TCTN3*) and OFDVI (caused by mutations in *TMEM216*, *TMEM231*, *TMEM138*, *C5orf42*, *TMEM107* and *KIAA0753*) [125,126].

*OFD1* gene encodes Oral-facial-digital syndrome 1 (OFD1) protein involved in formation of basal body and cilia [127]. OFD1 as a component of centrioles controls length of mother and daughter centrioles and recruits other proteins involved in ciliogenesis, including IFT88 or CEP164. Inactivation of *Ofd1* gene in mouse leads to progressive impairment of renal function [128].

### 2.9. Sensenbrenner Syndrome

Cranioectodermal dysplasia (CED) known as Sensenbrenner syndrome is characterized by sagittal craniosynostosis and facial, ectodermal and skeletal anomalies although some patients suffer from renal failure. CED is a rare disease with unknown exact frequency. So far more than 60 cases have been reported [129]. CED is caused by mutations in *IFT122*, *WDR35*, *IFT43* and *WDR19*, the encoding components of intraflagellar transport machinery. Functional analysis showed that *ift122* knockdown in zebrafish embryos leads to reduced number of basal bodies and cilia in the pronephric duct and shorter primary cilia of Kupffer vesicles. In human HEK293T cells *IFT122* knockout leads to cilia loss [129,130,131,132], while *Wdr35* knockout in mouse fibroblasts reveals strongly reduced level of IFT43 [133]. Human and mouse fibroblasts lacking *WDR35* fail to produce cilia [134]. WDR19 (IFT144) localizes mainly at the ciliary tip and at the base of cilium, while in Sensenbrenner syndrome patients it is absent or mislocalized [135].

All the above mentioned studies support the unifying theory of renal cystogenesis, coined more than 15 years ago, and further developed in later studies [17,136,137] According to this concept, mutations in genes expressed in primary cilia, basal bodies and centrosomes lead to dysfunction of cilia, thereby contributing to cell differentiation, stimulated proliferation and fluid secretion, as well as increased apoptosis of tubular cells, ultimately leading the development of cystic disease [17,136,137]. Further studies suggested that human ciliopathies could be caused by sorting defects at the transition zone and the ciliary gate [56]. It was initially proposed that the key molecular events contributing to cystogenesis are increased intracellular cAMP [17], or activation of MAPK/ERK and mTOR pathways [138,139,140]. However, studies in murine ADPKD models excluded the causative role of these signaling cascades and demonstrated that formation of cysts is rather triggered by other cilia-dependent pathways [138]. In accordance with this hypothesis, it was found that loss of cilia suppresses growth of cysts in mouse models with inactivated *Pkd1* or *Pkd2* [138]. According to this novel hypothesis, the unidentified ciliary signals called cilia-dependent cysts activation (CDCA) are inhibited by physiological actions of polycystins. Mutations affecting polycystin genes in ADPKD result in de-repression of CDCA, leading to aberrant proliferation of tubule cells, remodeling of base membrane and parenchyma and finally causing formation of cysts [141]. The specific molecular pathways involved in CDCA still await their identification, however, when discovered, may open new possibilities for treatment of ADPKD patients.

## 3. Relevance of Ciliary Genes in Renal Cancer

Renal cysts are characteristic feature not only of classic ciliopathies, but also of kidney cancer. Although cysts are present in a number of renal cancer subtypes, it is still controversial whether they may contribute to its development. Renal cell cancer (RCC), the most common subtype of malignant kidney tumors, is a collection of histologically and molecularly divergent subtypes, including clear cell RCC (ccRCC), papillary RCC (pRCC) and chromophobe RCC (chRCC) [142]. Most of RCC subtypes are characterized by loss of cilia [143,144], suggestive of their potential involvement in the neoplastic process. Indeed, the links between cilia and cancer have been found over 100 years ago when Theodor Boveri suggested that abnormal increase of centrosomes could lead to multipolar (abnormal) mitotic spindles and chromosomes instability, thereby contributing to cancer development [145]. Dependent mostly on Aurora A (AURKA) overexpression, centrosome amplification is present in variety of tumors. However, chromosomal instability may occur through many other proteins that regulate microtubules dynamics. For instance, depletion of *IFT88* (ciliary protein associated with kidney cyst) leads to accelerated cell cycle through the retinoblastoma tumor suppressor pathway [146]. Centrosome amplification results in formation of extra cilia what could have an impact on signaling output by contributing to disease phenotypes [127]. Furthermore, the loss of cilia may contribute to aberrant signaling by changing the insensitivity of cancer cells to environmental repressive signals such as cell cycle checkpoints [21]. In fact, development of renal cell cancer is associated with reduced ciliation [147]. The loss of cilia in the kidney releases basal bodies and provokes inappropriate cell division, aberrant Wnt, Hedgehog or PDGF signaling and overproliferation. Finally, strong evidence suggests that cilia have tumor suppressive effects controlling signaling pathways associated with tumorigenesis [148,149]. A recent study in mice triple mutant of tumor suppressors *Vhl*, *Trp53* and *Rb1* resulting in development of renal cancer, showed enrichment in ciliary genes mutations [147]. Furthermore, the same study demonstrated that 40% of the analyzed 448 human ccRCC tumors bear mutations in primary cilium-associated genes. The Cancer Genome Atlas (TCGA) data indicates that damaging mutations in *PKD1* and *PKD2* are found in a subset of tumors of various types. Moreover, ciliopathies and cancer share common regulatory pathways, including pro-proliferative signaling cascades activated by RTK, EGFR, HER2 receptors and their downstream effectors such as B-RAF, ERK, mTOR, AKT, SRC. Furthermore, similar patterns of upregulated transcription factors such as myc or c-fos are found in PKD and cancer [30,58]. It is thus tempting to think that ciliopathies and renal cancer might be regulated by the same ciliary genes. Table 1 includes examples of ciliary genes involved in cysts formation, renal failure and renal cancer.

### 3.1. The Role of VHL in Ciliogenesis and Renal Cancer

VHL encodes a multifunctional protein involved in the regulation of proliferation, apoptosis, senescence, extracellular matrix and cell responses to hypoxia [150]. Germline *VHL* mutations lead to von Hippel-Lindau disease, characterized by development of tumors in many organs, including the kidney. One of the well-known manifestations of von Hippel-Lindau disease is formation of cysts. It was suggested that VHL-loss induces formation of renal cysts which are precursor lesions that progress to clear cell renal cell carcinoma (ccRCC) in patients with von Hippel-Lindau disease [151]. However, most ccRCC cases arise as sporadic tumors for which development additional mutations in non-VHL loci are required [152].

VHL plays an important role in proper formation and maintenance of primary cilia of the kidney. pVHL is an E3 ubiquitin ligase that promotes proteasomal degradation of hypoxia-inducible factors (HIFs) that have been hydroxylated at their prolyl residues. Low oxygen levels prevent this posttranslational modification, restraining HIF-pVHL interactions and attenuating HIFs’ degradation at proteasomes. In consequence, the stabilized HIFs activate transcription of several target genes (e.g., VEGF, PDGF) that support cell’s adaptation to hypoxia. Mutations that inactivate VHL, lead to persistent HIF upregulation and activation of hypoxia-induced genes, regardless of the oxygen status [153].

VHL is also involved in functioning of cilia. pVHL localizes to the ciliary axoneme and basal bodies [154], and interacts with kinesin-2, a key protein mediating IFT [155]. Renal cancer cells devoid of active pVHL are cilia deficient [156], while re-expression of pVHL in RCC cells restores cilia in a manner dependent on HIF-1α degradation [157]. The VHL-mediated control of cilia formation is evolutionary conserved as vhl^−/−^ zebrafish mutants develop disorganized proximal pronephric tubules with disordered cilia [158]. Furthermore, pVHL protects microtubules from depolimerization *in vivo* [157] and pVHL-mediated stabilization of the axoneme microtubules is attenuated by GSK3β-mediated phosphorylation [159]. During cilia formation, pVHL controls the orientation of microtubules growing at cell periphery, in a mechanisms possibly involving a Par3–Par6–aPKC complex that regulates ciliogenesis [154]. According to the model provided by Kuehn et al. [156], pVHL-regulated ciliogenesis is tightly linked with cell cycle control. Specifically, pVHL binds to microtubules and the Par3-Par6-aPKC complex, which enables the migration of centrosome to the apical membrane. Following attachment to the transition fibers, the centrosome anchored to the membrane forms the basal body which enables the growth of cilium, which triggers signaling keeping the cell in G0 phase. In cells devoid of active pVHL, the formation of cilia is attenuated, releasing the cell cycle block and progression to mitosis [156]. Interestingly, it was shown that pVHL-dependent cilia formation enables kidney cells to mechanosense fluid flows, triggering rapid increase of intracellular Ca^2+^ concentration [155]. This model of VHL-cilia-mediated control of proliferation is supported by a recent study on pheochromocytoma (PCC) tumors [160]. Accordingly, loss of VHL associates with reduced ciliation and enhanced proliferation of PCC tumors cells, while disruption of cilia results in enhanced proliferation of PCC cells in vitro. Furthermore, cilia resorption in PCC is induced by hypoxia in a mechanism involving HIF1α and activation of AURKA/HDAC6 pathway. Interestingly, the same study showed that hypoxia reduces VHL presence in the ciliary axoneme [160]. In ccRCC cells, loss of VHL leads to stabilization and nuclear accumulation of β-catenin, which in turn activates transcription of AURKA, leading to activation of HDAC6, a tubulin deacetylase causing disassembly of microtubules that form the axoneme [161].

### 3.2. Other Ciliary Genes Involved in Renal Cancer

*NEK8* (never in mitosis gene A (NIMA)-related kinase 8) plays a critical role in DNA damage response/repair, cytoskeleton rearrangement and cilia formation. Its dysfunction is linked with polycystic kidney disease and several types of cancer [162,163,164,165,166]. It was suggested that proper maintenance of primary cilia structure in human renal cancer cells requires *NEK8* down-regulation in a mechanism involving pVHL and HIFs [167].

Altered DNA methylation of *NPHP4* was found in RCC subtypes. Specifically, *NPHP4* was hypermethylated in chromophobe RCC when compared with ccRCC, papillary RCC and renal oncocytoma samples, suggestive of its involvement in renal cancer development. *NPHP4* has been also associated with Wnt and Hippo tumor suppressor networks [168,169].

Tau Tubulin Kinase 2 (*TTBK2*) encodes a serine-threonine kinase which phosphorylates tau and tubulin proteins. *TTBK2* plays essential role for ciliogenesis initiation allowing cilia regrowth followed by exit from the cell cycle. It promotes CP110 removal from mother centriole and recruits IFT proteins to build the ciliary axoneme. *TTBK2* is a target for the development of novel strategies to overcome resistance to sunitinib in kidney carcinoma and melanoma-cell lines [170,171].

## 4. Ciliary Genes as Therapeutic Targets

The genes involved in cilia formation and functioning emerge as attractive targets for therapy. Cilia-related genes are frequently mutated in tumor cells, including colorectal or breast cancers [172,173]. Out of 304 known ciliary genes (published on the website of Syscilia project [174], http://www.syscilia.org/goldstandard.shtml), eight (*CTNNB1*, *DRD5*, *GSK3B*, *PLK1*, *SMO*, *TTK*, *VDAC3*, *VHL*) encode proteins that are targeted by compounds currently undergoing clinical trials (Table 2). The significance of cilia in ccRCC pathogenesis is strengthened by the observation that MLN4924, a compound suppressing cilia formation [175], attenuates proliferation and migration of ccRCC cells [176]. Furthermore, bexarotene, an RXR agonist, which restores ciliogenesis in ccRCC cells devoid of VHL in a mechanism involving AURKA reduction, attenuates incidence of ccRCC tumor xenografts in mice [177]. Ciliary proteins are also targeted in therapies of neurological disorders, including Alzheimer’s or Parkinson’s disease. Finally, several studies reported the potential of ciliary genes as diagnostic and prognostic markers of polycystic kidney disease (*PKD1*) and diverse disorders such as psoriatic arthritis (*NUP62*), non-small cell lung cancer (*TUBB3*) or acute kidney injury (*CLUAP1*) (Table 3). These promising results indicate that the significance of ciliary genes as biomarkers and therapeutic targets will be growing in future studies on the pathophysiology and treatment of ciliary diseases.

## 5. Conclusions

Formation of cysts is the common feature of renal ciliopathies and cancer. According to the unifying theory of renal cystogenesis, ciliopathies are caused by mutations in genes encoding proteins expressed in primary cilia, basal bodies and centrosomes which affect cilia functioning, thereby contributing to disturbed cilia-controlled signaling pathways. Strikingly, renal cancer is characterized by cilia dysfunction, linked with inactivation of its key tumor suppressor, *VHL*. There is a strong evidence that, similar to ciliopathies, loss of cilia in renal cancer cells contributes to enhanced proliferation. Furthermore, renal cancer and kidney ciliopathies share common mutated ciliary genes (e.g., *NPHPs* and *TMEMs*). All these studies suggest common cilia-related molecular mechanisms behind kidney ciliopathies and malignancy. Careful investigation of ciliary gene expression and mutation profiles may thus help in better diagnoses of renal pathologies. Successful clinical trials based on ciliary proteins antagonist or agonist provide evidence on a potential of these proteins as therapeutic targets in treatment of renal cystic diseases. Further extensive research is needed to understand the relationship between cilia, cilia-mediated signaling and renal cancer, and to reveal the details of pathogenesis of renal tumors and possible usage of ciliary proteins and their signaling partners as new targets for cancer therapeutics.

## Figures and Tables

**Table 1:** Cilia and renal cysts-associated diseases.

Diseases	Mutated Genes	Features
PKD	*PKD1*, *PKD2*	Renal cyst formation
Meckel-Gruber syndrome	*MKS1*, *TMEM67*, *TMEM216*, *TMEM107*, *TMEM231*, *CEP290/NPHP6*, *NPHP8/RPGRIP1L*, *CC2D2A*, *NPHP3*, *TCTN2*, *B9D1*, *B9D2*	Renal cyst formation
Nephronophthisis	*NPHP1*, *NPHP3*, *NPHP2 (INVS)*, *NPHP4*, *NPHP5/IQCB1*, *NPHP6/CEP290*, *NPHP7/GLIS2*, *NPHP8/RPGRIP1L*, *NPHP9/NEK8*, *TMEM67/MKS3*, *TTC21B/JBTS11*, *WDR19*, *ZNF423*, *CEP164*, *ANKS6*, *CEP83*, *DCDC2* *MAPKBP1*	Renal fibrosis and cyst formation
Joubert syndrome	*INPP5E*, *TMEM216*, *AHI1*, *NPHP1*, *NPHP6/CEP290*, *TMEM67/MKS3*, *RPGRIP1L/NPHP8*, *ARL13B*, *OFD1*, *TTC21B*, *TMEM237*, *CEP41*, *TMEM138*, *TCTN3*, *ZNF423*, *TMEM231*, *CSPP1*, *PDE6D*, *TCTN2*, *B9D1*, *MKS1*, *TMEM107*, *B9D2*, *ARL3*	Renal cyst formation
Oral-facial-digital syndrome type I	*OFD1*	Cystic kidney disease
Cranioectodermal dysplasia	*IFT122*, *WDR35*, *IFT43*, *WDR19/IFT144*	Renal failure
Bardet-Biedl	*BBS1*, *BBS2*, *ARL6*, *BBS4*, *BBS5*, *BBS6/MKKS*, *BBS7*, *BBS8*, *BBS9*, *BBS10*, *TRIM32*, *BBS12*, *CCDC28B*, *CEP290/NPHP6*, *TMEM67/MKS3*, *MKS1*, *SDCCAG8*, *LZTFL1*, *BBIP1*, *IFT27*, *IFT74*,	Chronic renal failure in children
Senior-Loken syndrome	*NPHP1*, *NPHP4*, *NPHP5*, *NPHP6*, *SDCCAG8*, *WDR19/IFT144*, *TRAF3IP1*	Juvenile nephronophthisis, renal cyst
Alström syndrome	*ALMS1*	Renal failure
RCC	*VHL*, *NPHP4*, *NPHP9/NEK8*, *TTBK2*	Cyst and cancer
Von Hippel-Lindau	*VHL*	Cyst and cancer

**Table 2:** Examples of ciliary genes use in cancer clinical trials (from TTD—Therapeutic Target Database http://db.idrblab.net/ttd/ (accessed on 6 November 2019) [178].

Ciliary Genes	Type of Cancer	Name of Drug	Clinical Trial	References
*CTNNB1*	Solid cancer	Recombinant human endostatin	approved	[179,180]
*DRD5*	Solid cancer	DS-8273	Phase1	https://clinicaltrials.gov/ct2/show/NCT02076451
*GSK3B*	Acute myeloid leukemia, osteosarcoma	LY2090314, Tideglusib	Phase2	https://clinicaltrials.gov/ct2/show/NCT01214603 [181]
*PLK1*	Solid cancer, Acute myeloid leukemia	Rigosertib, Volasertib	Phase3, Phase3	[182,183,184,185,186,187,188,189]
*SMO*	Solid cancer, skin cancer	LDE225 LY2940680, BMS-833923, LEQ-506, TAK-441	Approved (basal cell carcinoma) Phase1, Phase2	[190,191,192,193,194,195,196,197,198,199,200]
*TTK*	Solid cancer	BAY1161909, BAY1161909	Phase1 Phase1	[201,202,203]
*VDAC3*	Solid cancer	PRLX93936	Phase1/2	https://clinicaltrials.gov/ct2/show/NCT01695590
*VHL*	Renal cell cancer	Pyrrolidine carboxamide derivative 1	Patented-recorded Target	[204,205]

**Table 3:** List of ciliary genes as diagnostic, prognostic, theragnostic or associative biomarkers (from TTD—Therapeutic Target Database http://db.idrblab.net/ttd/ (accessed on 6 November 2019) [178].

Ciliary Genes	Biomarker Gene Location	Disease	Biomarker Type	Molecular Type	Biomarker Measure	Interactors	Reference
*PKD1*	16p13.3	Polycystic kidney disease	Prognostic	Gene	Mutation	-	[206]
*CLUAP1*	16p13.3	Acute kidney injury, Huntington disease	Diagnostic, Prognostic	Protein	Elevated level	-	[207,208]
*DRD1*	5q35.1	Hypertension	Associative	Gene	SNP	-	[209]
*DRD2*	11q23.2	Hypertension, schizophrenia	Associative, Theragnostic	Gene	SNP	-	[209,210]
*NUP62*	19q13.33	Psoriatic arthitis	Diagnostic	Gene	Expression	-	[211]
*PTCH1*	9q22.32-	Medulloblastoma	Theragnostic	Gene	Mutation	-	[212]
*SMO*	7q32.1-	Medulloblastoma	Theragnostic	Gene	Mutation	-	[212]
*TUBB3*	16q24.3	Non-small cell lung cancer	Prognostic	Protein	Absence	ERCC1	[213]

-: unknown.
